# A Smartphone App for Improvement of Colonoscopy Preparation (ColoprAPP): Development and Feasibility Study

**DOI:** 10.2196/mhealth.7703

**Published:** 2017-09-20

**Authors:** Benjamin Walter, Roland Schmid, Stefan von Delius

**Affiliations:** ^1^ Universitätsklinik Ulm Medizinische Klinik I Universität Ulm Ulm Germany; ^2^ Klinikum rechts der Isar der Technischen Universität München II. Medizinische Klinik und Poliklinik Technische Universität München München Germany

**Keywords:** bowel preparation, smartphone app, intestinal cleansing, patient education, colonoscopy, colonoscopy preparation

## Abstract

**Background:**

Optimal bowel preparation is one of the major cornerstones for quality of colonoscopy. But poor bowel preparation still occurs in 10% to 25% of all patients. To optimize patient guidance, we developed a new smartphone app (ColoprAPP) for Android smartphones which guides and accompanies the patient starting 4 days before colonoscopy throughout the whole colonoscopy preparation procedure.

**Objective:**

The objective of this study was to assess the function of a newly developed smartphone app for supporting colonoscopy preparation.

**Methods:**

We carried out a prospective feasibility study including 25 patients undergoing outpatient colonoscopy at our hospital. As a control, we retrieved the data of 25 patients undergoing outpatient colonoscopy matching in age, sex, and indication for colonoscopy from our colonoscopy database. Patients were asked to download the smartphone app, ColoprAPP, in addition to being given the regular colonoscopy preparation leaflet. All colonoscopies were performed in the morning after using a split-dose preparation containing a polyethlene glycol–based purgative. The study was designed to test feasibility of the prototype, evaluate grade of bowel cleanliness (Boston bowel preparation scale [BBPS]), and assess patient satisfaction with the app.

**Results:**

The smartphone app use was feasible in all patients. BBPS count as a marker for grade of bowel preparation was significantly higher in the smartphone app–supported group (mean 8.1 [SD 0.3] vs 7.1 [SD 0.4], *P*=.02). Left (mean 2.8 [SD 0.1] vs 2.4 [SD 0.11], *P*=.02) and transverse colon (mean 2.8 [SD 0.07] vs 2.4 [SD 0.11], *P*<.001) revealed significantly higher BBPS counts in the smartphone app–supported group than in controls. Patient satisfaction with a smartphone app–supported colonoscopy preparation was high with an average numeric rating scale score for usefulness of 8.2 (visual analog scale 1-10).

**Conclusions:**

A novel developed smartphone app for reinforced education of bowel cleansing was feasible and led to high BBPS scores and patient satisfaction.

**Trial registration:**

ClinicalTrials.gov NCT02512328; https://clinicaltrials.gov/ct2/show/NCT02512328 (Archived by WebCite at http://www.webcitation.org/6sz3Kk26z)

## Introduction

Optimal bowel preparation is a major cornerstone of colonoscopy quality [1]. Improvement of bowel preparation by split-dose purgative regime has already raised the quality [2]. Nevertheless, bowel preparation is still inadequate in a considerable amount of patients [3]. Many factors have already been described like socioeconomic and educational reasons [4]. Education and continuous guidance of patients has been shown to ensure quality of colonoscopy preparation and patient compliance. Numerous approaches using additional booklets, cartoons, educational videos, and text messaging have shown a direct benefit [5-8]. Nevertheless, all approaches still have their specific limitations toward the availability of the medium.

Worldwide, an enormous increase in the use of smartphones has taken place. Integration of this new medium could help optimize the preparation procedure. We therefore created a novel smartphone app to improve colonoscopy preparation. The primary purpose of the actual study was then to evaluate if this newly developed app for Android smartphones is feasible for colonoscopy preparation. Secondary endpoints were patient satisfaction and bowel cleanliness.

## Methods

### Smartphone App Development

**Figure 1:**
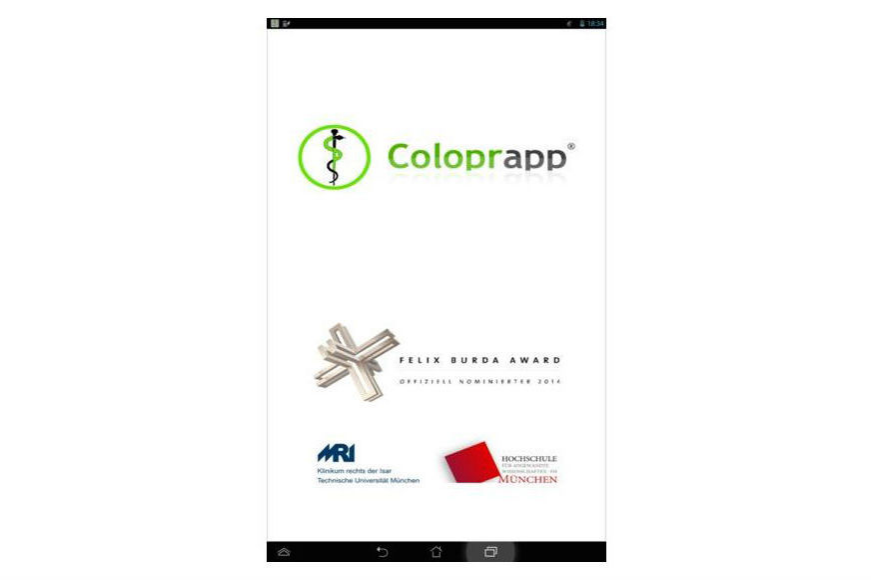
Start desktop of ColoprAPP.

**Figure 2:**
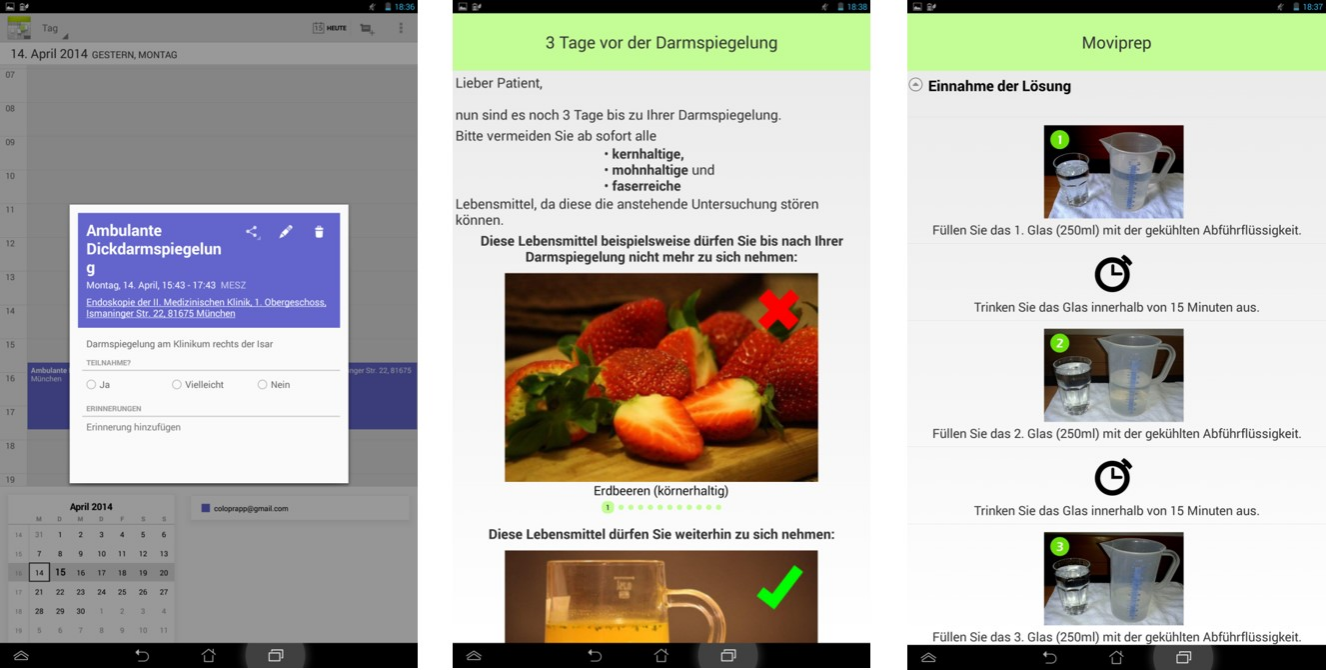
Screenshot examples of app function, calendar function (left), dietary recommendations (middle), and purgative instructions (right).

The Colonoscopy Preparation App (ColoprAPP) is an app (Figures 1 and 2) for mobile devices using the leading mobile operating system, Android. ColoprAPP is written in the official development language for Android called Java. The app does not need access to the Internet after download. Focus is on data security (more details about software development in Multimedia Appendix 1). The smartphone app was developed in cooperation with the University of Applied Sciences, Munich.

After the smartphone app was developed, this prospective study was conducted at the II. Medizinische Klinik, Klinikum rechts der Isar, Technische Universität München. The study protocol was approved by the local ethics committee. Informed consent was obtained from the study participants. The study is registered at ClinicalTrials.gov [NCT02512328].

### Colonoscopy Preparation Scheme

At our hospital, the colonoscopy preparation standard is a regular polyethylene glycol–based split-dose regime (MoviPrep, Norgine SA). Explanations of the preparation procedure are given during the informed consent discussion several days prior to the endoscopy. Furthermore, a leaflet containing detailed diet and preparation recommendations is given to every patient undergoing colonoscopy. For quality assurance reasons, colonoscopy preparation is routinely measured by the Boston bowel preparation scale (BBPS) (≥5 points as the threshold for sufficient bowel cleansing) [9].

### Feasibility Study Performance

A total of 25 patients undergoing outpatient colonoscopy were included in the smartphone app group. Inclusion criteria were outpatient colonoscopy, written informed consent, aged >18 years, smartphone owner (Android), German (national) mobile phone provider. Exclusion criteria were, due to feasibility study design, very strict excluding patients with phenprocoumon therapy, diabetes mellitus with insulin therapy, pregnancy, recent neurologic illnesses, reported previous electrolyte disturbances, and smartphones with other operating systems than Android (eg, iOS, Microsoft).

The smartphone app was immediately downloaded, saved, and activated from a cloud software storage to the patient’s smartphone after study inclusion. All colonoscopies took place in the morning, and 3 in-house endoscopists performed the colonoscopies. They did not get any information on participation status in app-supported colonoscopy preparation.

At the time of colonoscopy, a questionnaire was given to the smartphone app study group subjects asking for (1) history of prior colonoscopies (experience in bowel preparation), (2) when smartphone app was downloaded and used, (3) extent of discomfort caused by preparation procedure, (4) whether the smartphone app was perceived as helpful, (5) whether smartphone app was perceived a hindrance toward preparation, (6) whether the patient would favor the use of smartphone app-supported colonoscopy preparation again, and (7) whether patients would recommend the smartphone app to friends or family members.

Satisfaction with the smartphone app was assessed using a numeric rating scale (NRS) from 1 to 10 (1=not helpful to 10=very helpful; counterparted: 1=not inhibitory to 10=very inhibitory).

For reasons of comparison, BBPS data of 25 patients from our outpatient colonoscopy database matching inclusion criteria and in age, sex, first colonscopy or not status, and indication for colonoscopy who underwent outpatient colonoscopy at our institution during the study period were used as “matched pairs.” In case of more than one match, the one with a higher BBPS was chosen.

### Statistical Analysis

A total of 25 patients were planned to be included in the feasibility study. As a control, data from 25 patients matching in age, sex, and indication were taken from the local endoscopic database. Descriptive statistics were computed for all variables to provide means and standard deviations for continuous variables and frequencies for categorical variables. Total BBPS scores were calculated (smartphone app study group and controls). *P* values correspond to the Mann-Whitney U test, Student *t* test with Welch correction, and the paired *t* test. A *P* value <.05 was considered statistically significant. The results for colon preparation were dichotomized to adequate preparation (BBPS total score 5-9) and inadequate colon preparation (BBPS total score <5). All statistical analyses were performed with the statistical software Graphpad Prism (Graphpad Software Inc).

**Table 1:** General patient characteristics.

Characteristic		n or mean
**Sex, n**		
	Male	11
	Female	14
Age, years, mean (SEM^a^)		44.1 (13.0)
**Age, years, mean**		
	Male	49.4
	Female	40.0
**First colonoscopy, n**		
	Yes	14
	No	11

^a^SEM: standard error of the mean.

**Table 2:** Indications for colonoscopy performed.

Indication for colonoscopy	n (%)
Colon cancer screening	11 (44)
Unclear abdominal pain	8 (32)
Inflammatory bowel disease (not first diagnosis)	4 (16)
Other	2 (8)

## Results

### Patient Characteristics

For the study, a total of 25/25 patients with outpatient colonoscopy were included. Male/female ratio 1:1.3 (11 male, 14 female). Mean age of participants 44.1 (SD 13.0) years (male: 49.4 years, female: 40.0 years) (Table 1). Indications for colonoscopy were colon cancer screening (11/25, 44%), unclear abdominal pain (8/25, 32%), inflammatory bowel disease (not first diagnosis) (4/25, 16%), other (2/25, 8%); 14/14 patients were having first colonoscopy, and 11/11 had already one or more colonoscopies in their medical history (Table 2). Data of matched pairs were taken from colonoscopy database at the hospital. Pairs matched in age, sex, indication for colonoscopy, and previous colonoscopies.

### Primary Endpoint

The smartphone app prototype was sufficiently working with stable function during the time period of colonoscopy preparation in all 25 participants. Programmed information for procedure and recommendations were provided to the participants in a correct time-adjusted manner.

### Secondary Endpoints

**Figure 3:**
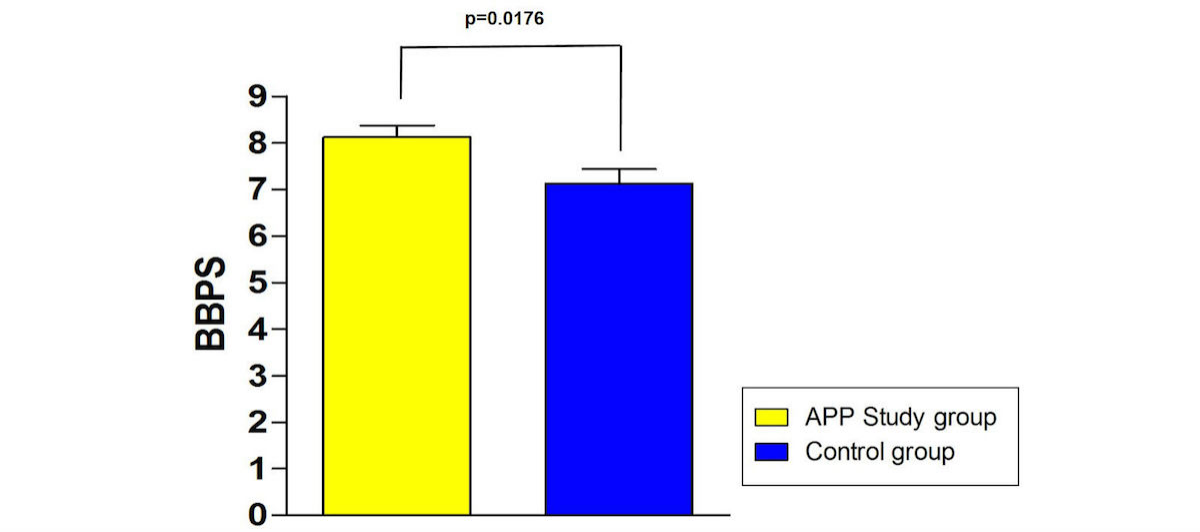
Total Boston bowel preparation scale score with significantly higher grade for smartphone app–supported group.

**Figure 4:**
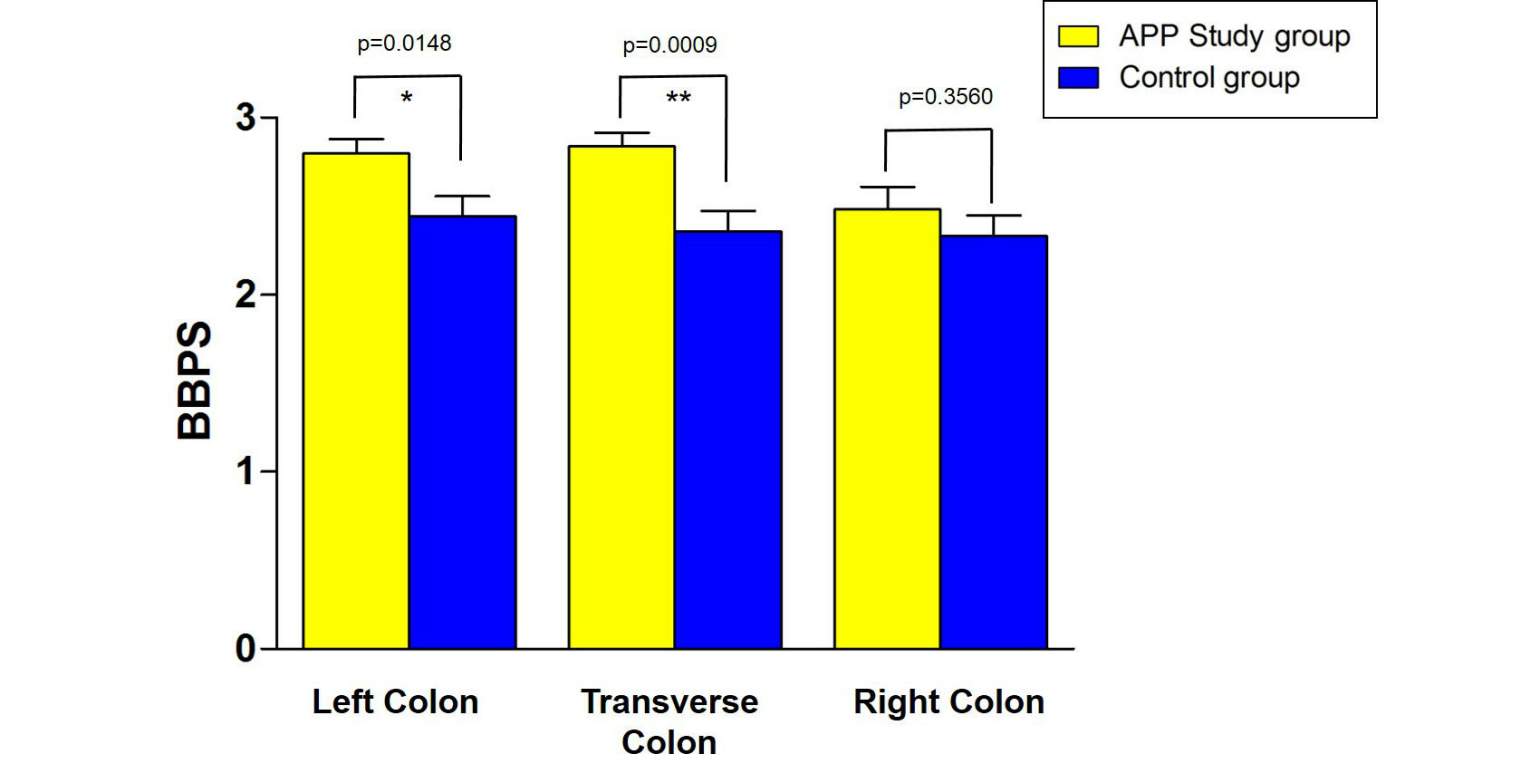
Average Boston bowel preparation scale score split into colonic regions (left, transverse, and right colon) with a significantly higher score for left and transverse colon.

Mean BBPS score of the smartphone app study group was 8.1 (SD 0.25) versus 7.1 (SD 0.41) (*P*=.02 for difference) (control group) (Figure 3). The mean BBPS score of the smartphone app study group of the left colon was 2.8 (SD 0.08) versus 2.4 (SD 0.11) (*P*=.02). The mean BBPS score of the smartphone app group of the transverse colon was 2.8 (SD 0.07) versus 2.4 (SD 0.11) (*P*<.001). The mean BBPS score of the smartphone app group of the right colon was 2.5 (SD 0.13) versus 2.3 (SD 0.11) (*P*=.36) (Figure 4). With regard to an insufficient bowel preparation (BBPS <5), there was 1 patient in the control group, whereas all smartphone app study participants achieved a BBPS ≥5.

All study participants of the smartphone app group would use the smartphone app again and recommend the system to their friends and relatives. When asked if the smartphone app was helpful to get the colonoscopy preparation done, patients reported an average NRS score for usefulness of 8.2. The smartphone app was not found to be a hindrance by an average NRS score for inhibitory effect of the smartphone app of 1.4 (25/25). Preparation for colonoscopy in general was reported to be unpleasant by an average NRS score of 6.1.

## Discussion

### Principal Findings

Poor bowel preparation is associated with a bundle of problems and causes an even more reduced acceptability of colon cancer screening programs [10], so this remains a concern. To reach an optimal bowel preparation, close patient guidance and education are crucial [11]. Previous published studies evaluated different forms of interventions and media to improve patient education [1,2,5,6,8].

Smartphones may have an even higher impact for close and up-to-date guidance for our patients, as many people carry a smartphone 24 hours a day. Smartphone apps are software platforms for smartphones which could be easily used for patient education. To date there is little research on the effect of smartphone apps for colonoscopy preparation. But data already published (only for iOS) seem to be very promising [12].

### Smartphone App Development

We designed a free-of-charge, offline working smartphone app (ColoprAPP) for Android smartphones with the objective to improve patient satisfaction and bowel preparation for colonoscopy. The app offers various educational tools and gives reminders on time-axis adjusted instructions for all steps of colonoscopy preparation (Figures 1 and 2).

### Feasibility Study

This study showed that using a smartphone app is feasible to support colonoscopy preparation and could be an add-on to improve patient education. Use of the app improved grade of bowel cleanliness when compared to matched controls without reinforced educational measures. The participants perceived the smartphone app service as very helpful and would recommend its use to friends and relatives.

### Study Limitations

This feasibility study is subject to some limitations. First, it had only a small number of participants, and second, the app was only available for Android-based smartphones. We note that matched pairs for comparison can include some bias. The use of smartphones varies by different factors including age, sex, and socioeconomic factors, which by themselves influence the quality of bowel preparation. The real impact of a smartphone app–based reminder system on the quality of bowel cleansing has therefore to be shown in larger randomized studies. We also understand that statistics are very limited due to the small number of study participants, and therefore conclusions regarding improvement of bowel cleanliness are also very limited. Results could only be interpreted as a tendency.

### Conclusion

We conclude that a newly developed Android-based smartphone app for reinforced education to improve colonoscopy preparation is feasible. It works offline to maximumize patient data safety. The provided 4 days of guidance containing dietary recommendations and recommendations for laxative intake supported patients to get through the colonoscopy preparation.

## Appendix

Multimedia Appendix 1 Technical details about the smartphone app.

## Abbreviations

BBPS: Boston bowel preparation scale
ColoprAPP: Colonoscopy Preparation App
NRS: numeric rating scale
