# Supplementary material for: A Smartphone App for Improvement of Colonoscopy Preparation (ColoprAPP): Development and Feasibility Study
Source: JMIR Mhealth Uhealth. 2017 Sep 20;5(9):e138. doi: 10.2196/mhealth.7703 (PMC5628282; doi:10.2196/mhealth.7703)
Supplement: Multimedia Appendix 1 [file mhealth_v5i9e138_app1.pdf]

***Supplement 1***

***Development***

For the development of ColoprAPP© the agile software development methodology Scrum was applied. The development team used the Integrated Development Environment Eclipse and TortoiseSVN (over Dropbox) as development tools for versioning, whereas Dropbox acted as Subversion-Server to enable easy access for the distributed developers on the repository. After each function was implemented, the function was tested and the Code was submitted to the repository. Therefore, at any point of the development process, executable code was available in the repository and immediate feedback of the team members and the product owner on the new functionality was made possible.

In order to test the business logic, the team implemented several tests written in the framework Junit. Different mobile devices like Samsung Galaxy S, Samsung Galaxy S2, Samsung Galaxy S3 mini, HTC One, and Asus MeMo Pad were used for end user tests.

***Architecture***

ColoprAPP© consists of a typical three layer architecture – the presentation layer, application layer and the data access layer. To enable loose coupling and separation of concerns the architectural design pattern MVC was applied.

Presentation layer: All UI-Elements like layouts, colors, fonts, font sizes and text elements are defined in extensible markup language files, to enable independence of UI Elements from Java-Code and to preserve changeability and flexibility with minimal efforts. As the ColoprAPP© supports small screens like those of Smartphone and large screens like those of Tablets, different layouts where defined to achieve the best usability depending on the screen size (responsive layer).

The business layer mainly consists of an event engine, which calculates the notifications for the preparation steps depending on the planned date of the colonoscopy and therefore enables the app to act as a coach for the user. Depending on the point in time of the preparation process, different information is provided to the user, such as information regarding the complete process of the colonoscopy including the preparation and the follow up, allowed and forbidden food and beverages as well as information about the trip, back and forth, to the doctor.

The data access layer enables the business layer to access saved data. As ColoprAPP© doesn’t need to save a huge amount of data, the App uses the very simple, key-value based approach called Shared Preferences instead of a SQLite database.

To ensure user anonymity and to be independent of continuous internet connection the SPA works offline and autonomously without server data exchange after download. The SPA is free of charge.

The patient can choose to look up short or more extense informations about colonoscopy preparation. An educational cartoon about relevance of colon cancer screening provided is also included (COMIC "So simple, so easy - Colonoscopy preparation", *Felix Burda Foundation, Munich, Germany)*.
